# Microplastics in cardiopulmonary bypass: quantification and characterization of particles across systems

**DOI:** 10.1093/icvts/ivaf080

**Published:** 2025-06-10

**Authors:** Jordan L Green, Daniel T Field, Robert Bennett, Lauren C Jenner, Emma C Chapman, Laura R Sadofsky, Jeanette M Rotchell, Mahmoud Loubani

**Affiliations:** Centre for Cardiology and Cardiothoracic Surgery, Hull University Teaching Hospitals NHS Trust, Castle Hill Hospital, Hull, UK; Hull York Medical School, University of Hull, Hull, UK; Centre for Cardiology and Cardiothoracic Surgery, Hull University Teaching Hospitals NHS Trust, Castle Hill Hospital, Hull, UK; Hull York Medical School, University of Hull, Hull, UK; Centre for Cardiology and Cardiothoracic Surgery, Hull University Teaching Hospitals NHS Trust, Castle Hill Hospital, Hull, UK; Hull York Medical School, University of Hull, Hull, UK; Hull York Medical School, University of Hull, Hull, UK; School of Natural Sciences, University of Hull, Hull, UK; Hull York Medical School, University of Hull, Hull, UK; School of Natural Sciences, University of Hull, Hull, UK; Centre for Cardiology and Cardiothoracic Surgery, Hull University Teaching Hospitals NHS Trust, Castle Hill Hospital, Hull, UK; Hull York Medical School, University of Hull, Hull, UK

**Keywords:** microplastic, polymer, cardiopulmonary bypass, minimally invasive extracorporeal circulation (MiECC), FTIR

## Abstract

**OBJECTIVES:**

This study determines the microplastic (MP) levels, dimension, shape, and chemical composition generated from conventional cardiopulmonary bypass and minimally invasive extracorporeal circulation (MiECC) circuits.

**METHODS:**

*In vitro* conventional and MiECC circuits, mimicking realistic setups with 2 L of Hartmann’s solution were run for 90 min (*n* = 3 circuit runs each), filtered, and analysed using micro-Fourier transform infrared spectroscopy alongside procedural blanks (*n* = 5).

**RESULTS:**

Conventional circuits produced 60.4 ± 7.6 MPs L^−1^h^−1^ (77.0% of the total particles). MiECC circuits produced 48.4 ± 31.3 MPs L^−1^h^−1^ (45.3% of total particles). MP levels in each circuit type were significantly elevated compared with procedural blank (*n* = 5) samples (5.6 ± 10.4 MPs L^−1^h^−1^) but did not differ with respect to the other. Twenty different MP polymer types were detected whereby polydimethyl siloxane, poly(decyl methacrylate), and poly(N-butyl methacrylate) represented the most MPs within conventional circuits. For MiECC, the most abundant were polypropylene, polyethylene, and polyamide. Average MP lengths differed significantly: 93.5 ± 98.6 µm (conventional) versus 62.0 ± 54.4 µm (MiECC) (*P *< 0.001), although widths did not differ. Film particles (48.2%) were the predominant shape for conventional circuits and fragments (50.5%) for MiECC.

**CONCLUSIONS:**

Significant levels of MP particles were produced across the two systems. Future studies can determine the time points at which they are produced in machine use, to mitigate their production, as well as inform cell/tissue culture investigations into the clinical significance of their introduction into patients undergoing cardiac surgery.

## GRAPHICAL ABSTRACT

**Figure:**
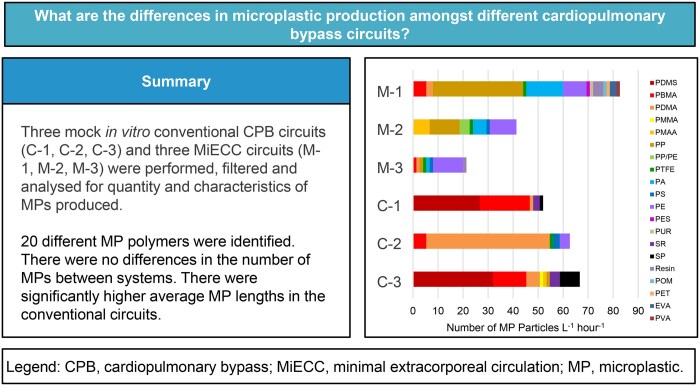

## INTRODUCTION

Microplastic (MP) studies within environmental settings highlight their ubiquitous nature in various media including air, aquatic, and terrestrial environments [1–3] and recently, in human samples [4–6]. The term MP is generally defined as plastic debris between 1 and 1000 µm in size [7]. Plastic consumption within healthcare is high, with the medical plastics market worth £16 billion worldwide [8]. Items such as gloves, packaging, and blood tubes/syringes are the largest contributors within the UK National Health Service, and it is estimated that 133 000 tonnes of plastic are disposed of each year [8]. The examination of plastics in terms of the characterization of their debris particles including polymer types, sizes and shapes, has not been performed within cardiac surgery to date.

From 2009 to 2018, more than 280 000 operations were conducted within cardiac surgery in the UK [9], with the majority involving cardiopulmonary bypass (CPB). CPB is an extracorporeal machine that takes over the function of the heart and lungs [10]. CPB includes a venous line taking blood away from the patient into a reservoir, an oxygenator, and an arterial line returning blood to the patient. Blood is circulated via a roller pump design where positive displacement via the deformation of the plastic circuit tubing creates movement [11]. Minimally invasive extracorporeal circulation (MiECC) has a design that minimizes the length of tubing (omitting the suction device and cardiotomy reservoir), the circulating volume is reduced, and blood flow is produced via centrifugal force [12].

Past studies have reported that plastic particles, within the MP size range, are produced within mock CPB circuits [13, 14]. Their presence was attributed to the spallation of polyvinyl chloride (PVC) tubing that is in contact with the roller pump of the circuit. Forces that deform the tubing are hypothesized to cause fractures and sheering of plastic source chemicals from the tubing’s inner lining into the fluid within the CPB circuit [15]. Particles of 1 nm to 500 µm have been detected in *in vitro* circuits [16], yet detailed characterization is limited.

Recent studies provide insight into the impacts of MPs using human cell/tissue-based experiments [17] and rodents [18–20]. One finding is that MP shape, levels and exposure duration all influence the detrimental outcomes measured as immune response and oxidative stress end-points [21]. It is well understood that cardiac surgical patients are at increased risk of cardiac, respiratory, renal, and cerebrovascular morbidity [21], and it is possible that exposure to MP products generated from CPB may clinically correlate with these complications. Yet, the quantification and characterization of these MPs and the ultimate clinical effects within cardiac surgery, if any, are not yet established. This study aimed to determine the particle dimensions, shapes, and chemical composition of MPs that patients are exposed to from two different designs of CPB circuits.

## MATERIALS AND METHODS

### Study design

This study was exempt from ethical approval as this does not involve participants or use animal/human tissue. Three *in vitro* conventional (labelled C-1, C-2, C-3) and three *in vitro* MiECC (labelled M-1, M-2, M-3) circuit runs were used to investigate the generation of particles in circulating solutions. The CPB circuit components were manufactured and supplied by Liva Nova PLC (London, UK) with components within the circuits coated with *Phisio* (phosphorylcholine). The conventional circuit consisted of a membrane oxygenator (Sorin Inspire 6F M, Liva Nova PLC), a hard-shell venous reservoir (Sorin Inspire HVR Dual, Liva Nova PLC), a roller pump (^1^/_2_″ silicone plastic pump boot), two roller pump suckers (^1^/_4_″ silicone plastic pump boots), and a ^3^/_8_″/½″ PVC tubing loop connecting the arterial outlet to the venous inlet. In comparison, the MiECC circuit consisted of a membrane oxygenator (Sorin Inspire 6F M, Liva Nova PLC), a bubble trap (Inspire VBT 8, Liva Nova PLC), a centrifugal pump (Sorin Revolution, Liva Nova PLC), a softshell reservoir (Sorin BMR 1900) used to prime the circuit, and a ^3^/_8_″ PVC tubing loop connecting the arterial outlet to the venous inlet.

A Stockert SIII Heart Lung Machine (Liva Nova PLC) was used to drive the pumps and measure circuit pressures. Both circuits were primed with 2 L Hartmann’s solution (LOT 21H11T36, Baxter Healthcare Ltd, Thetford, UK) containing 5 ml (5000 IU) heparin (LOT 5002351, CP Pharmaceuticals Ltd, Wrexham, UK). After priming and de-airing, the Hartmann’s solution (2 L) was re-circulated via the PVC tubing loop at 4 L/min for 90 min representing a clinically average time for a bypass operation. Circuit pressure was maintained at 115 mmHg by clamping the venous inlet. A heater-cooler unit (Maquet HC40, Maquet Getinge Group, Rastatt, Germany) was connected to the heat exchanger of the oxygenator to maintain the temperature of the circulating solution at 37°C. For the conventional circuit two roller pump suckers continuously removed and returned prime solution to and from the venous reservoir at 400 ml/min (to mimic roller pump suction used in conventional bypass circuits). For the MiECC the softshell reservoir was used for priming and was clamped off during the 90 min recirculation. A type III MiECC circuit was used in this study. All circuits were run on the same day, in the same environment, and were operated by the same two researchers. After 90 min, each CPB circuit was turned off, and the full 2 L circulating volume was drained into pre-prepared glassware, covered with foil, and then transported to the laboratory for analysis.

### QC and QA procedures

Strict control measures were used to characterize unavoidable background MP contamination, from solutions or air, as outlined previously [7, 22]. Five procedural blanks were conducted. For the two circulation solutions used: 6 L of Hartmann’s solution and one 5000-unit vial (5 ml) of the unfractionated heparin were filtered and analysed. A separate, pre-cleaned, 1 L empty glass beaker (*n* = 1) was placed within the CPB room to characterize airborne MPs. Glass beakers were pre-cleaned by hand washing, dishwashing with distilled water for 30 min at 70°C and washing three times with triple filtered MilliQ water. A foil lid was used to cover the beakers.

### Sample collection procedure

All samples were collected in pre-cleaned glass beakers and filtered using an Anodisc 47 mm 0.2 µm pore size aluminium oxide filter (Anodisc inorganic filter membrane, Whatman, Buckinghamshire, UK) as described in Field *et al.* [23]. No standardized protocols exist within the international MP research field to account for background contamination, so two approaches were adopted: subtraction and limit of detection (LOD)/limit of quantification (LOQ) approach [24]. Using the raw data, subtraction of blanks, and LOD/LOQ adjusted results provides a comparison using each technique (Supplementary Table S1).

### Particle chemical composition analysis

Measurements were conducted with a µFTIR spectrometer (model Nicolet iN10, ThermoFisher, Waltham MA, USA) in liquid nitrogen-cooled transmission mode with machine settings as described previously [23]. Resulting sample spectra were compared to polymer libraries (Omnic Picta, Omnic Polymer Libraries) and full spectral ranges were used with a match threshold of ≥70% (termed hit quality index threshold) (Supplementary Fig. S1). The total number of particles identified from the filters in the circuits (operated for 1.5 h, three replicates for each of the two machine types) above the 70% hit quality index threshold was 459, for which 245 (53%) were characterized as MPs. Only one quarter of each filter was analysed using μFTIR, therefore results were multiplied by 4 to represent the whole filter as it was assumed that MPs were distributed evenly across these.

### Statistical analysis

Data are presented as mean ± standard deviation. Statistical analyses were performed using IBM SPSS Statistics Version 27 (IBM SPSS Software, Chicago, USA). Following appropriate normality testing, it was determined that non-parametric statistical methods should be used; the Kruskal–Wallis or Mann–Whitney *U* test were therefore used, where appropriate. A *P*-value of less than 0.05 was considered statistically significant.

## RESULTS

### Procedural blank particle levels and characterization

The MP levels detected in the procedural blank (*n* = 5) samples were 5.6 ± 10.4 MPs L^−1^h^−1^. The laboratory filtration step blanks (*n* = 2) contained only non-MP particles, consisting of zein (80%) and cellulose (20%). Similarly, six 1 L bags of Hartmann’s solution contained no MPs and a level of 11.33 ± 3.01 non-MP particles L^−1^, mainly zein (58.8%). The heparin solution contained 4800 MP particles L^−1^, of six MP polymer types: epoxy- and phenoxy-resins, polydimethylsiloxane (PDMS), poly(4-chlorostyrene): styrene, polytetrafluoroethylene (PTFE) and 1,7-dichlorooctamethyltetrasiloxane relative to 19 200 L^−1^ of non-MP particles of mostly zein (38%) and cellulose/cellophane (17%). The number of MPs detected within the CPB room (for the sampling period, following conversion to standard units of m^−2^d^−1^) was 5051 MP m^−2^d^−1^, with one MP polymer, polyphthalamide, represented. The bulk of total particle levels (∼97 000 m^−2^d^−1^) were non-MPs zein (44%) and cellulose (44%).

### Total particles detected within circuits

**Figure 1:**
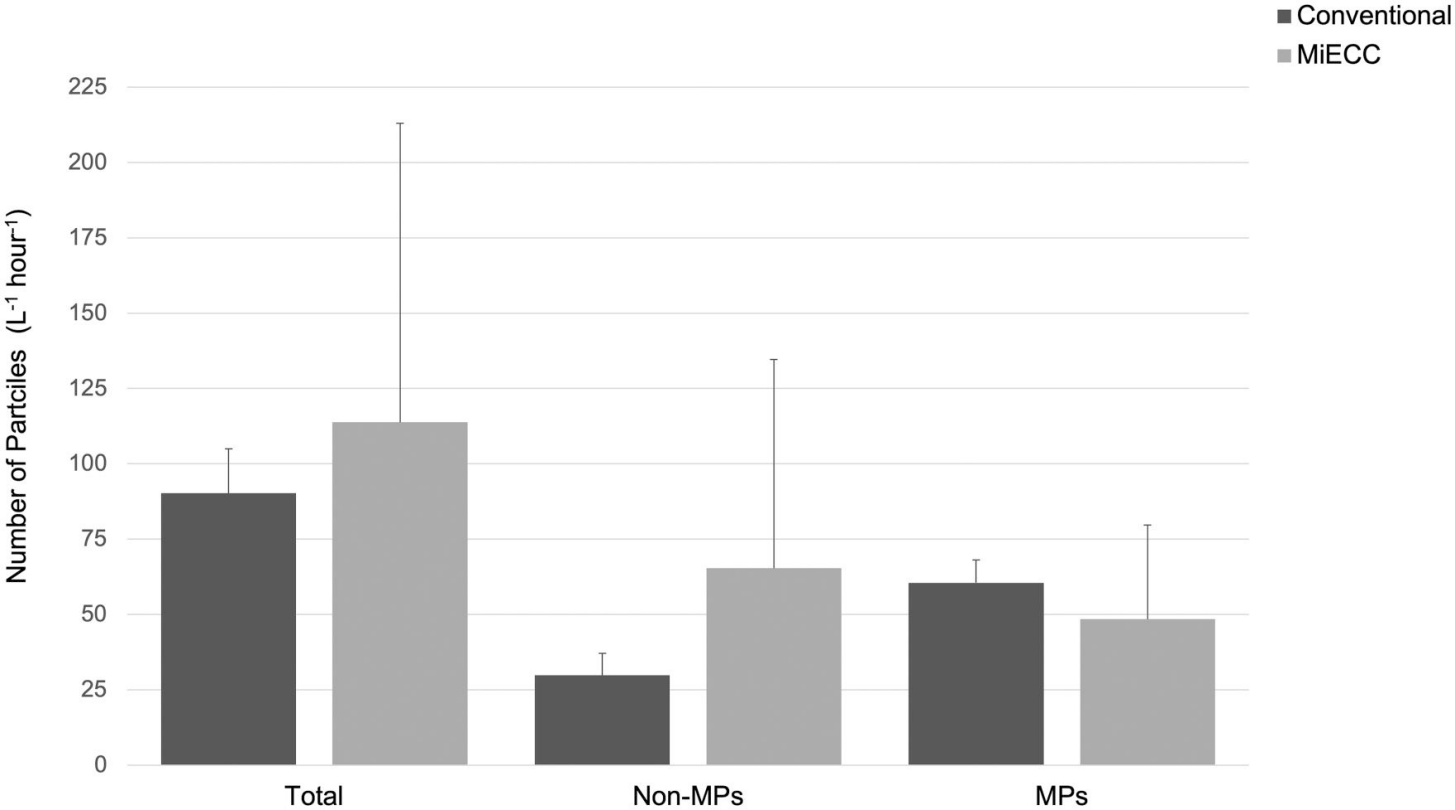
Particle production (mean and standard deviation) across the conventional cardiopulmonary bypass and MiECC circuits (*n* = 3 for each type of circuit). No significant differences were observed. MiECC, minimally invasive extracorporeal circulation; MP, microplastic

Mean total particle levels detected across all circuits was 102.0 ± 64.7 particles L^−1^h^−1^. Within the conventional circuits, the total particle levels varied, with a mean 90.2 ± 14.7 particles L^−1^h^−1^ and a range of 73.3–100.0 particles L^−1^h^−1^ (Fig. 1). Within the MiECC circuits, there was a mean total level of 113.8 ± 99.2 particles L^−1^h^−1^, with a range of 49.3–228.0 particles L^−1^h^−1^. There was no significant difference in total particle levels detected between circuits (*P *= 1.00). Non-MP particles detected represented 33.0% of the conventional circuit particles (29.8 ± 7.3 particles L^−1^h^−1^), whereas this represented 54.7% of the MiECC circuit particles (65.3 ± 69.3 particles L^−1^h^−1^). No significant difference was observed in the total non-MP particle levels detected between circuits (*P *= 0.700).

### MP levels detected

**Figure 2:**
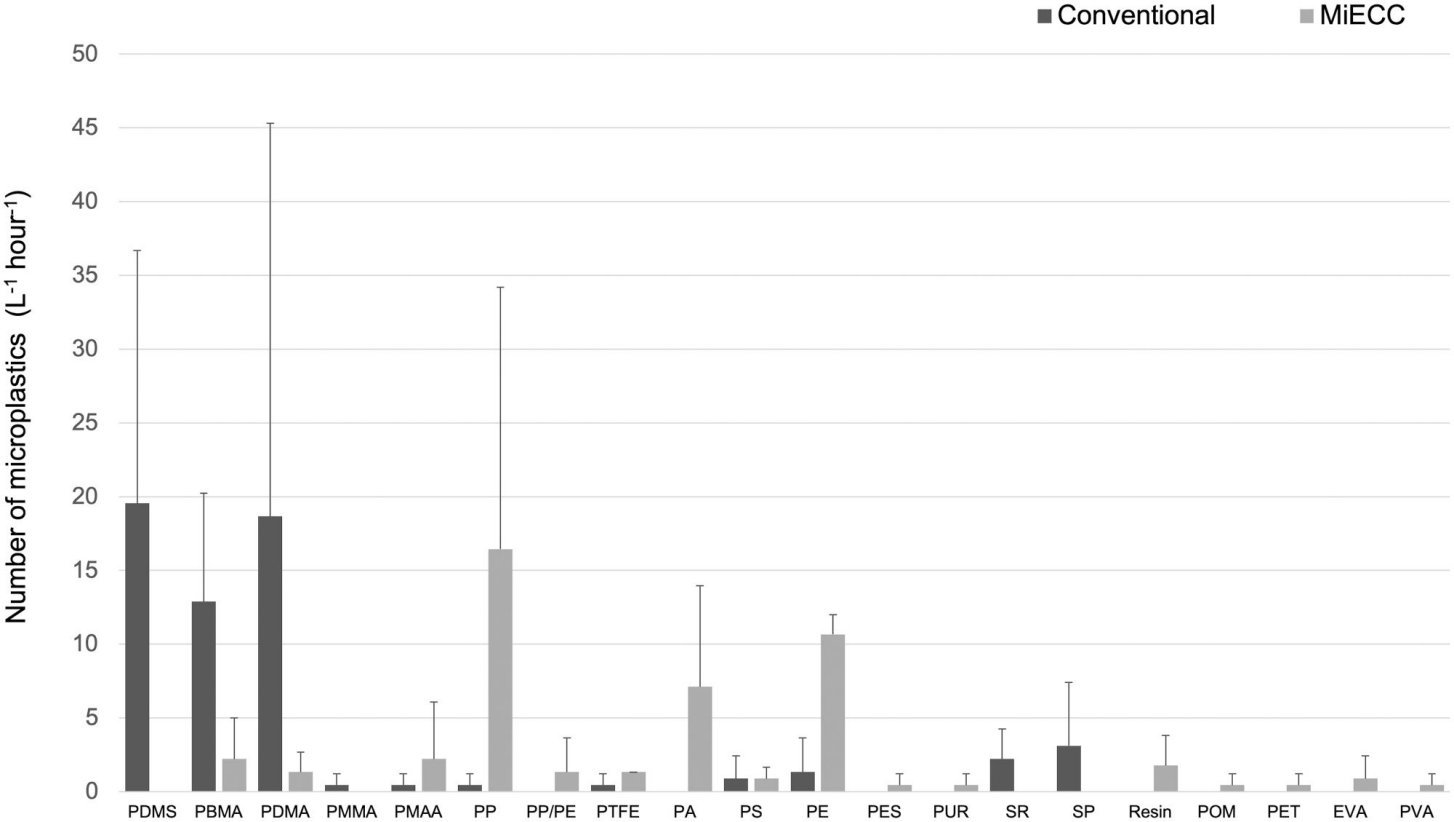
MP production (mean and standard deviation) across the conventional cardiopulmonary bypass (CPB) and MiECC circuits (*n* = 3 for each), characterized by MP polymer type. MiECC, minimally invasive extracorporeal circulation; MP, microplastic; PDMS, polydimethylsiloxane; PBMA, poly(N-butyl methacrylate); PDMA, poly(decyl methacrylate); PMMA, polymethyl methacrylate; PMAA, poly(N-methyl acrylamide); PP, polypropylene; PP/PE, polypropylene polyethylene copolymer; PTFE, polytetrafluoroethylene; PA, polyamide (nylon); PS, polystyrene; PE, polyethylene; PES, polyester; PUR, polyether urethane; SR, silicone rubber; SP, silicone polymer; POM, polyacetal; PET, polyethylene terephthalate; EVA, polyethylene vinyl acetate; PVA, polyvinyl acetate

Conventional circuits produced 60.4 ± 7.6 MPs L^−1^h^−1^, representing 77.0% of the total particles identified (Fig. 2, Supplementary Table S1). In comparison, MiECC circuits produced 48.4 ± 31.3 MPs L^−1^h^−1^, representing 45.3% of the total particles identified. There was no significant difference between the levels of MPs produced between the two different circuits (*P *= 1.00) though the levels differed significantly for both circuits (both *P*s = 0.017) relative to the procedural control samples.

Twenty different MP particle types were detected. Polydimethylsiloxane (PDMS), poly(decyl methacrylate) (PDMA) and poly(N-butyl methacrylate) (PBMA) accounted for the greatest proportion of MP production for conventional circuits, with a mean of 19.6 ± 17.1, 18.7 ± 26.6 and 12.9 ± 7.3 particles L^−1^h^−1^, respectively. Polyethylene (PE), silicone rubber (SR), silicone polymer (SP), polymethyl methacrylate (PMMA), poly(N-methyl acrylamide) (PMAA), polypropylene (PP), polytetrafluoroethylene (PTFE), and polystyrene (PS) particles were also generated by the conventional circuits in lesser numbers. In contrast, within the MiECC circuit runs combined, the most abundant MPs were PP, PE, polyamide (PA), with a mean of 16.4 ± 17.8, 10.7 ± 1.3, and 7.1 ± 6.8 particles L^−1^h^−1^, respectively. Despite being the most abundant MP generated within the conventional circuits, PDMS was not detected within the MiECC circuits; moreover, PDMA and PBMA were generated within the MiECC circuits but in much smaller numbers compared to the conventional circuits. Other MPs generated within the MiECC circuits were PMAA, PP/PE copolymer, PTFE, PS, polyester (PES), polyether urethane (PUR), resin, polyacetal (POM), polyethylene terephthalate (PET), polyethylene vinyl acetate (EVA), and polyvinyl acetate (PVA). The total levels of each MP were not significantly different between the two circuit types (*P *> 0.05).

### MP characterization

**Figure 3:**
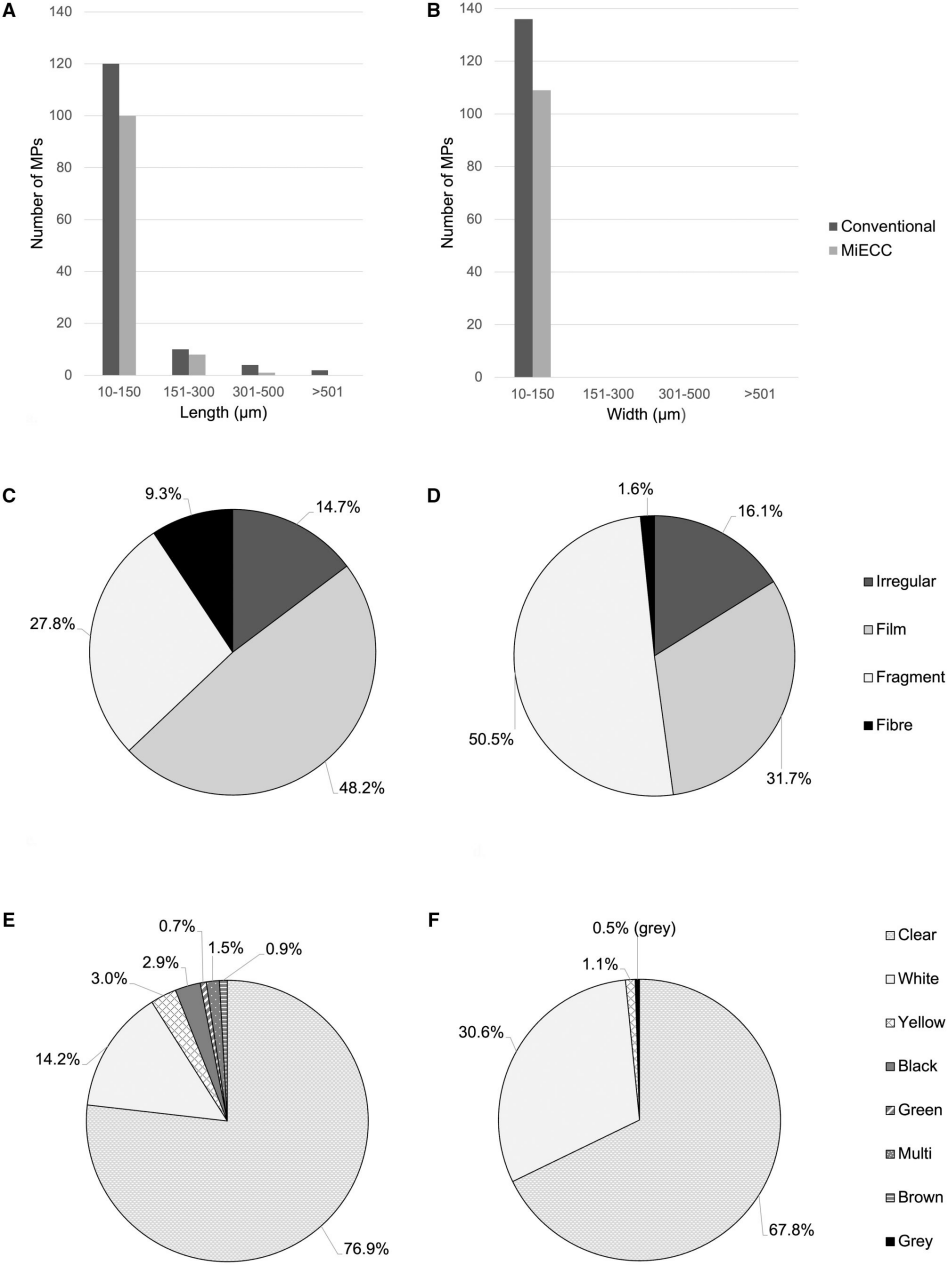
MP particle characteristics. MP lengths/widths (**A**/**B**) from conventional and MiECC circuits combined; MP shapes for (**C**) conventional circuits and (**D**) MiECC circuits; MP colours for (**E**) conventional circuits and (**F**) MiECC circuits. MiECC, minimally invasive extracorporeal circulation; MP, microplastic

In terms of MP particle dimensions, there was a significantly different (*P *< 0.001) mean MP length of 93.5 ± 98.6 µm versus 62.0 ± 54.4 µm for the conventional and MiECC circuits, respectively (Fig. 3A). Mean MP widths, which did not differ significantly, of 31.3 ± 18.1 µm versus 28.0 ± 15.8 µm, respectively, were observed for the two different circuit types (Fig. 3B). The procedural blanks displayed a mean MP length of 49.9 ± 18.6 µm, and MP width was 27.1 ± 11.6 µm, not significantly different from either circuit MP size dimensions.

Within the conventional circuits, films (48.2%) were the main shape generated followed by fragments (27.8%), fibres (9.3%) and irregular (14.7%) particles, respectively (Fig. 3C). In contrast, fragments (50.5%) represented the predominant particle shape generated within the MiECC circuit, followed by film (31.7%), irregular (16.1%) and fibre (1.6%) particles, respectively (Fig. 3D). The procedural blank MPs were mainly irregular shapes (57.1%) followed by fragments (28.6%) and film (14.2%).

The most abundant colour of MPs across the circuits was clear, representing 76.9% and 67.8% of the conventional and MiECC circuits as well as 57.1% in the procedural blank samples (Fig. 3E). This was followed by white, occupying 14.2% of the conventional circuits and 30.6% of the MiECC circuits. Small quantities of other coloured MPs were observed in the circuits (Fig. 3F).

### Adjustments

Using adjustments, to account for blank contamination levels detected, decreases the level of MPs identified within circuit samples depending on the approach used (Supplementary Table S1). After blank (regardless of polymer type) subtraction adjustments, the total MPs identified within conventional circuit samples have a mean of 58.58 ± 4.11 MPs L^−1^h^−1^. The total MPs identified within MiECC circuit samples have a blank adjusted mean of 46.58 ± 27.81 MPs L^−1^h^−1^. Using the LOD/LOQ calculation approach, focussing only on the most abundant MP particles detected across the two bypass circuits, the following polymers fit the criteria. For the conventional bypass circuits in turn: C-1 fit the criteria for using both LOD and LOQ calculation in several polymer types: PDMS, PBMA, PDMA, SR, SP, PP and PMAA, with a range of 1.3–31.7 MPs L^−1^h^−1^. C-2 only fit the LOD/LOQ criteria for three polymer types: PBMA, PDMA and PE with a range of 4–5.3 MPs L^−1^h^−1^. C-3 fit the LOD/LOQ criteria PDMS, PBMA, SR and SP with a range of 2.7–26.3 MPs L^−1^h^−1^. Fewer polymer types fit the LOD/LOQ criteria in the MiECC circuits: 4 polymers PBMA, PDMA, PP and PE in M-1 and M-3 with ranges of 1.3–12 and 2.7–36 MPs L^−1^h^−1^, respectively. M-2 had the least polymer types detected above the quantification threshold level, as PP, PE and PMAA with a range of 6.7–12 MPs L^−1^h^−1^.

## DISCUSSION

This study is the first to quantify and characterize MP production into the circulating fluids within conventional and MiECC CPB circuits using a robust quality assurance (QA)/quality control (QC) methodology. The MP levels detected were: 60.4 ± 7.6 MPs L^−1^h^−1^ and 48.4 ± 31.3 MPs L^−1^h^−1^ within the conventional and MiECC circuits, respectively. Twenty different MP types were detected but the predominant MP polymer differed with PDMS > PDMA > PBMA within conventional circuits, and PP > PE > PA within MiECC circuits. There was also a significant difference (*P *< 0.001) in the MP size (lengths but not widths)/shape characteristics between the two circuits with longer (∼94 µm) film-shaped particles in the conventional circuits compared with shorter lengths (∼62 µm) of fragment-type particles in the MiECC circuits, characteristics that are linked with detrimental outcomes in human cell studies [17].

In terms of clinical implications for a patient undergoing a 3-h operation on a CPB machine, it is possible to extrapolate to a MP contamination level worst-case scenario as follows: by assuming even particle spallation from machinery parts throughout machinery operation, taking the three most abundant polymer types only for each circuit, and assuming a surgery duration of 3 h, a total of 283 PDMS, PBMA and PDMA film-shape particles, of a mean length of ∼93 µm, would be introduced into the circulating fluids with a conventional CPB circuit. In comparison 164 PP, PE, PMAA fragment-shape particles, with a mean length of 62 µm, would be introduced into the circulating fluids using the MiECC circuit. The implications of the introduction of hundreds of MPs, which are durable and do not biodegrade, into the blood of CPB patients are not known. Yet, there is evidence that irregular MPs trigger immune system (inflammation and oxidative stress) responses in human cell-based experimental scenarios [17]. It has also been suggested that micro-emboli observed in deaths following CPB surgery have been caused by fragments (of ∼10 µm size) of silicone antifoam agents (rather than PVC tubing also tested) [25].

**Figure 4:**
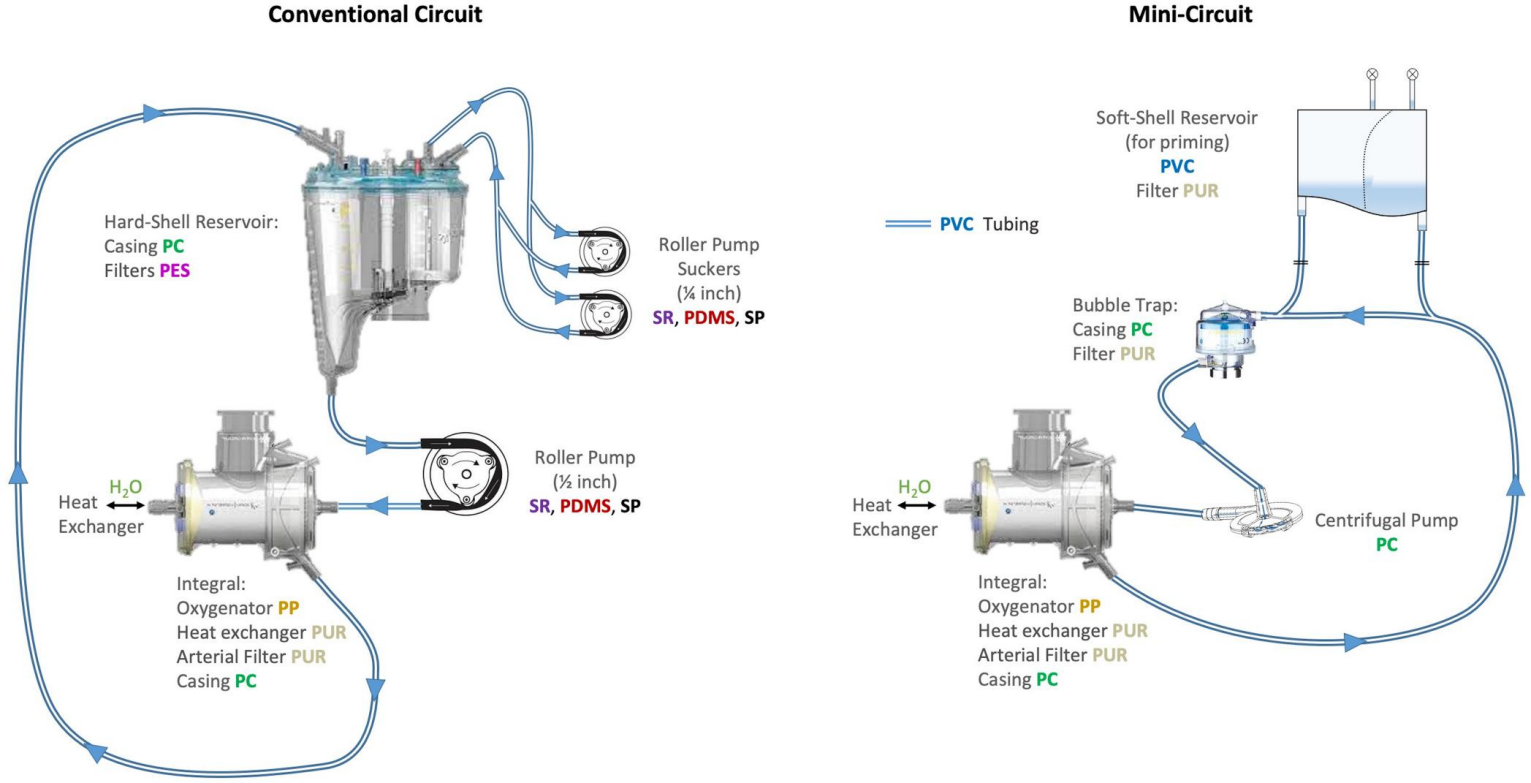
Schematic diagram of the conventional and MiECC circuits. Potential sources of MPs are highlighted. PBMA, poly(N-butyl methacrylate); PP, polypropylene; PC, polycarbonate; PE, polyethylene; PUR, polyether urethane; PVC, polyvinyl chloride; SR, silicone rubber; SP, silicone polymer

Having established that some of the MP particles identified could be spallation products from the CPB machine operation, their possible source requires discussion (Fig. 4). PDMS levels were high in the conventional circuits relative to the MiECC circuits. These are used as a shatterproof alternative to glass as well as to make filters for continuous renal replacement therapy for patients undergoing CPB and post-operatively to prevent post-perfusion syndrome. The hard-shell reservoir (not present in the MiECC circuits) may represent a potential source of these MPs. PP can be used as an alternative to silicone-based polymers when configuring membrane oxygenators to be used in CPB [26]. PP levels were the highest of any MP polymer in the MiECC circuits, potentially from the softshell reservoir used for priming, though was also detected at smaller levels in the conventional circuits. PE and PA particles were found at high levels in the MiECC circuits. PE oxides have been explored as a surface coating of hollow fibre membrane oxygenators based on their potential ability to reduce coagulation, inflammation markers and influence clinical outcomes [27].

Spallation of tubing has been suggested as the source of particles, with Tygon (a multi-polymer type) or silicone the main two sources[18]. Silicone has been demonstrated to have worse spallation performance than Tygon, via degradation of tubing both at compression sites from the roller pump and in the circuit itself [28] (Fig. 4). Both SR and SP, and PDMS, were detected at high levels in the conventional bypass circuits compared to the MiECC circuits. This is consistent with a study of particles generated [28] and with sources of silicone-based MPs in the conventional system compared to the MiECC system, which has no roller pumps (Fig. 4). Published studies support the notion that the longer the roller pump is active the more particles are produced [14, 16, 28]. Another theory is that the machinery produces more particles at the beginning of the operation during warm-up. Another important consideration is the inner tube diameter and wall thickness and how that changes the spallation of MPs [16]. The current understanding of plastic particle production within CPB is that spallation occurs at the roller pump [14, 16, 28]. MiECC systems do not use roller pumps; therefore, it is theorized they will produce fewer MP particles via spallation. Nevertheless, we suggest to mitigate unnecessary introduction of MPs into circulating fluids there should be machinery pre-runs to flush out any MPs generated. Moreover, the utility of pre-bypass filters has not been investigated in this context and therefore may represent an alternative method of reducing MP generation.

The clinical relevance of these findings is not yet known; though studies have sought to understand the impact of MPs in human cells and surrogate animal tissues. It has been found that polystyrene MPs (PS-MPs) within rat cardiomyocytes cause oxidative stress, leading to apoptosis and pyroptosis through increased expression of interleukins 1β [18]. *in vitro* studies have linked PS-MPs in pulmonary tissues with cytotoxic, inflammatory and barrier effects within normal human lung epithelial cells, increasing the risk of lung disease [19]. Studies using human embryonic kidney 293 cells have suggested that PS-MPs adhere to the cell membrane and lead to cytotoxicity [20]. Moreover, laboratory animal studies report that MPs cause abnormalities in the cardiovascular system, including vascular inflammation, myocardial damage, and increased blood pressure [29], with the underlying mechanisms unknown. In this specific patient group, post-operative cardiac, respiratory, and renal complications are seen in a significant proportion of those undergoing procedures involving CPB, thus it is plausible that this is a contributing factor to post-operative morbidity. Further studies addressing the mechanisms in which clinical effects are exhibited are needed.

A crystalloid solution, rather than a blood-based solution, was used to mimic realistic CPB setups within this study. Due to the evolving nature of this research field, an established method of analysing MPs within blood samples is yet to be agreed upon, with emerging studies presenting how this may be accomplished in different settings [4]. Notably, Hua *et al.* (NCT 05600010) have designed a study evaluating MPs in blood and cardiac tissue in an attempt to address this. We, therefore propose future research to investigate the direct effects of using human blood within CPB circuits, using this pilot data as a foundation, now that we have demonstrated the presence of MPs within different CPB circuits.

There are limitations to highlight. First, a crystalloid solution was re-circulated at a constant flow, pressure, and temperature, yet cardiac surgery involves fluctuations more likely to affect MP release. Second, the inner surfaces of the CPB circuits may be contaminated with debris during the manufacturing process and the MPs detected represent an initial ‘wash out’. A robust methodology for MP detection using procedural blanks detected a low level of background MP contamination, consistent with the recent study of MPs in an operating theatre, where 1924 ± 3105 MP m^−2^day^−1^ were reported [23]. The QA/QC approach used accounts for these by application of the LOD/LOQ calculation. Another limitation is that only one quarter of each filter was analysed and only two models of *in vitro* bypass circuits were modelled, limiting the inferences of the statistical analyses conducted. Moreover, the time-dependent relationship between MP production and CPB run-time was not investigated. Lastly, it is important to recognize that the number of circuits used was small, with no sample size calculations performed. This is due to the practical limitations and large quantity of research time needed to analyse such samples. Moreover, the purpose of this small sample size study was to generate a proof-of-concept dataset that will inform future larger studies.

## CONCLUSION

To the best of our knowledge, this is the first study to characterize and differentiate MP production between conventional and MiECC systems. Both contribute to MP generation with varying levels of MP polymer types across the two systems, yet there were significantly longer MPs produced in the conventional circuits. Using these findings, it is now possible to identify the sources of entry into CPB circuits by matching identified MPs to component polymers in the two circuits, enabling future research to understand how CPB design may be modified to mitigate risk. Moreover, this study provides directed rationale for targeted tissue studies using the polymer types identified herein, permitting investigations into the true clinical significance of MPs generated by CPB.

## Supplementary Material

ivaf080_Supplementary_Data

## Data Availability

The raw data are available on request to the corresponding author.

## ABBREVIATIONS

CPB: Cardiopulmonary bypass
EVA: Polyethylene vinyl acetate
MiECC: Minimally invasive extracorporeal circulation
MP: Microplastic
PA: Polyamide (nylon)
PBMA: Poly(N-butyl methacrylate)
PC: Polycarbonate
PDMA: Poly(decyl methacrylate)
PDMS: Polydimethylsiloxane
PE: Polyethylene
PES: Polyester
PET: Polyethylene terephthalate
PMAA: Poly(N-methyl acrylamide)
POM: Polyacetal
PP: Polypropylene
PP/PE: Polypropylene polyethylene copolymer
PS: Polystyrene
PS-MP: Polystyrene-microplastic
PTFE: Polytetrafluoroethylene
PUR: Polyether urethane
PVA: Polyvinyl acetate
PVC: Polyvinyl chloride
SP: Silicone polymer
SR: Silicone rubber
