# Supplementary material for: Microplastics in cardiopulmonary bypass: quantification and characterization of particles across systems
Source: Interdiscip Cardiovasc Thorac Surg. 2025 Jun 10;40(6):ivaf080. doi: 10.1093/icvts/ivaf080 (PMC12161987; doi:10.1093/icvts/ivaf080)
Supplement: ivaf080_Supplementary_Data [file ivaf080_supplementary_data.zip › Supplementary Figure SF1.docx]

**Supplementary Figure 1.** Selected images of the MPs identified within circuit samples alongside the spectra obtained (i) poly-dimethyl siloxane (PDMS), (ii) poly decyl methacrylate (PDMA), (iii) silopren, (iv) polyethylene (PE).

Wavenumbers (cm^-1^)

% Transmittance


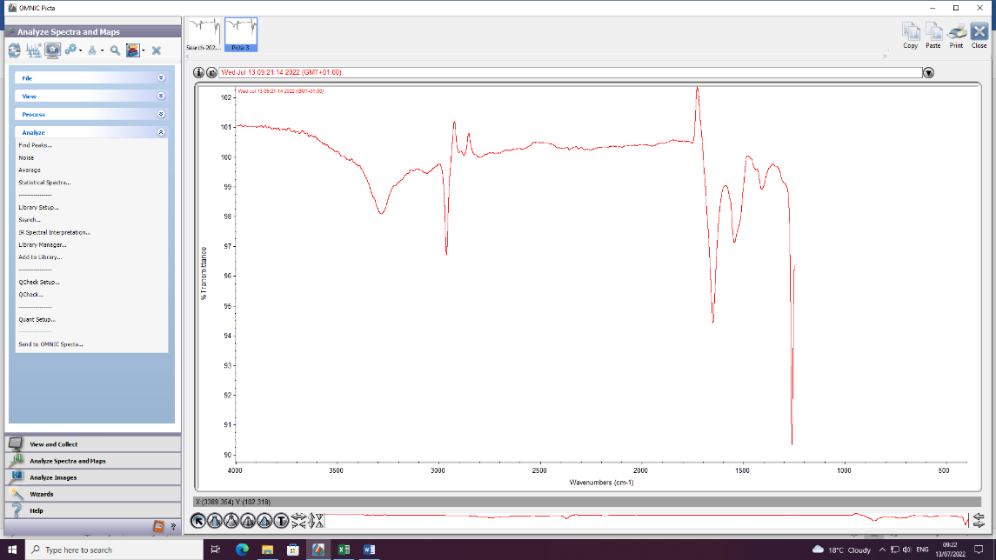


1000

2000

3000

4000

100

92

90

94

98

96

102


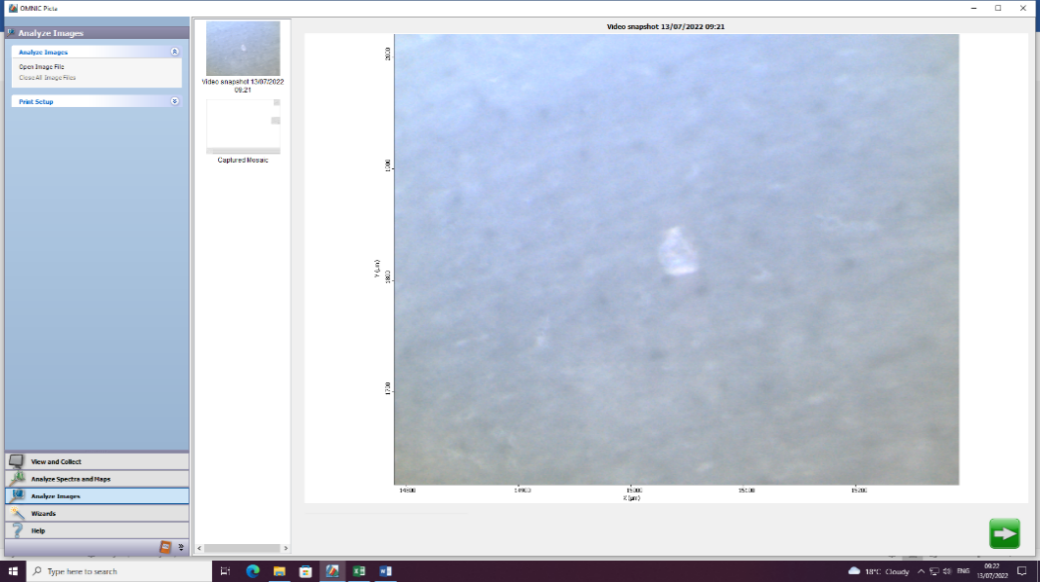


100 μm

(i)9i

% Transmittance

Wavenumbers (cm^-1^)


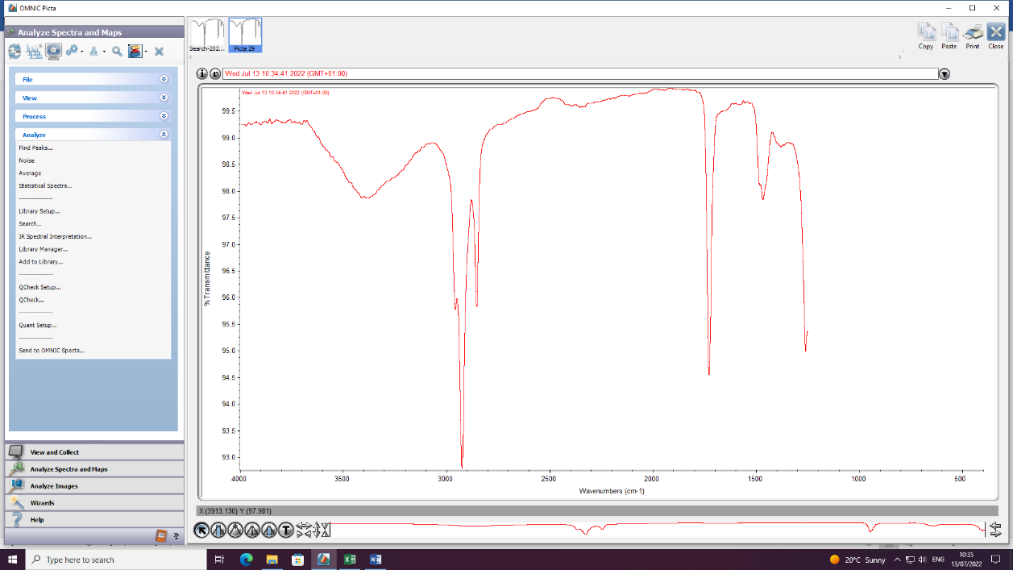


1000

2000

3000

4000

99

97

95

93


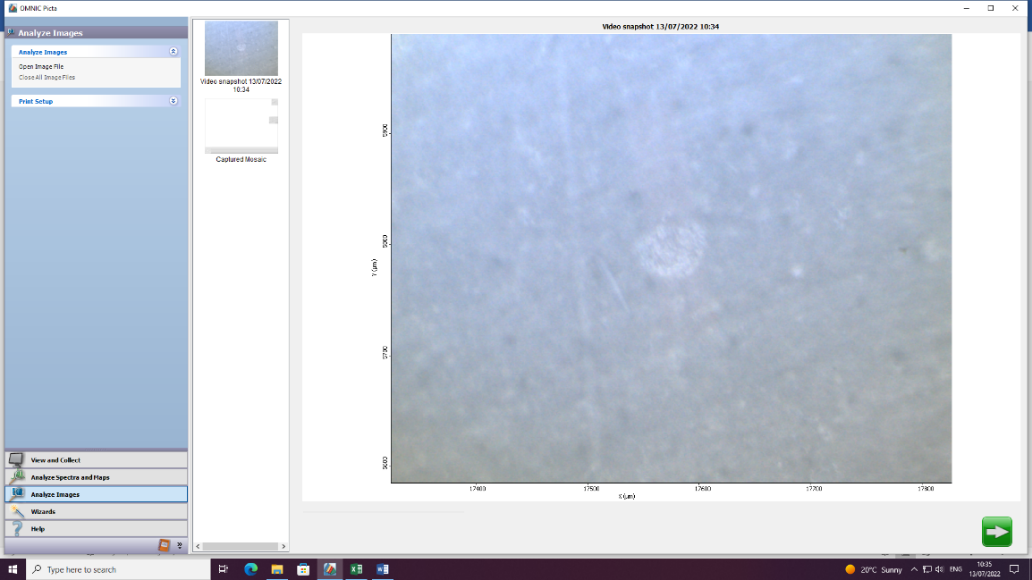


100 μm

(ii)


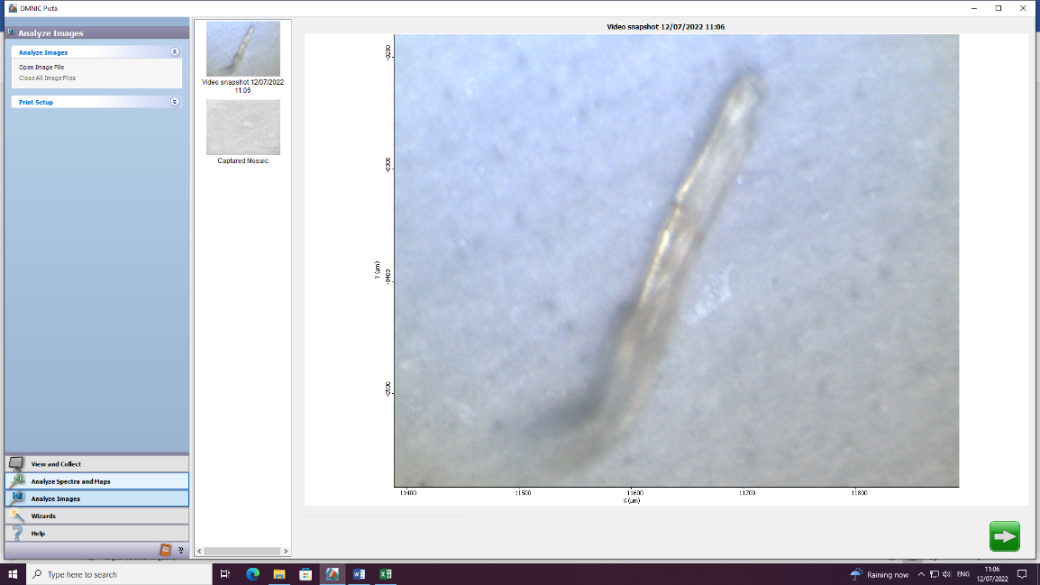


100 μm

(iii)

% Transmittance

Wavenumbers (cm^-1^)


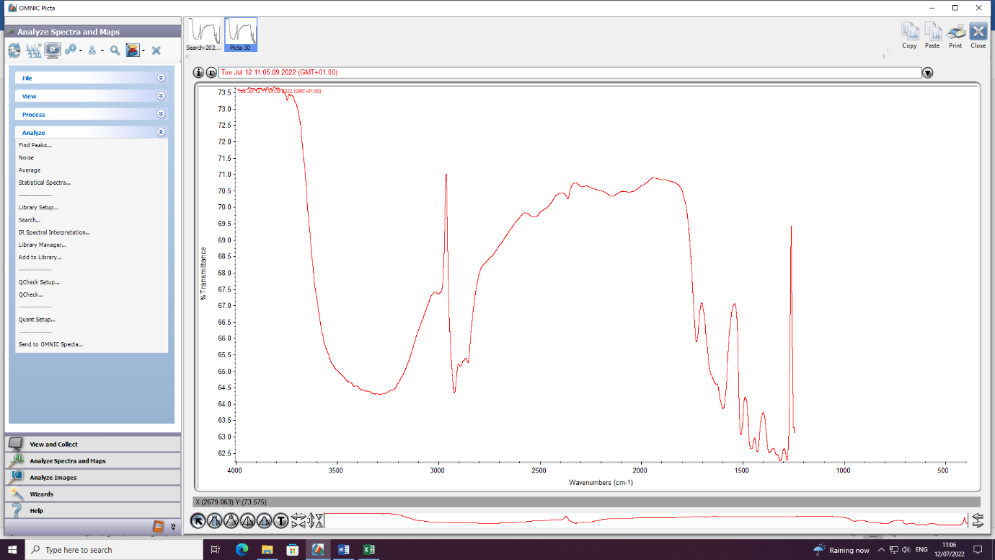


1000

3000

4000

200011

70

68

66

64

72


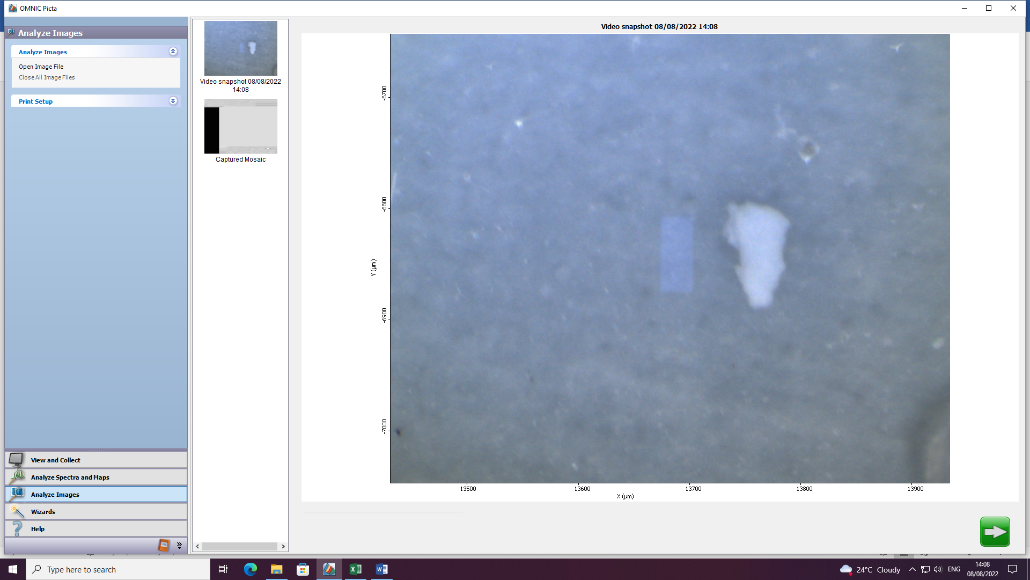


(iv)

100 μm

% Transmittance

Wavenumbers (cm^-1^)


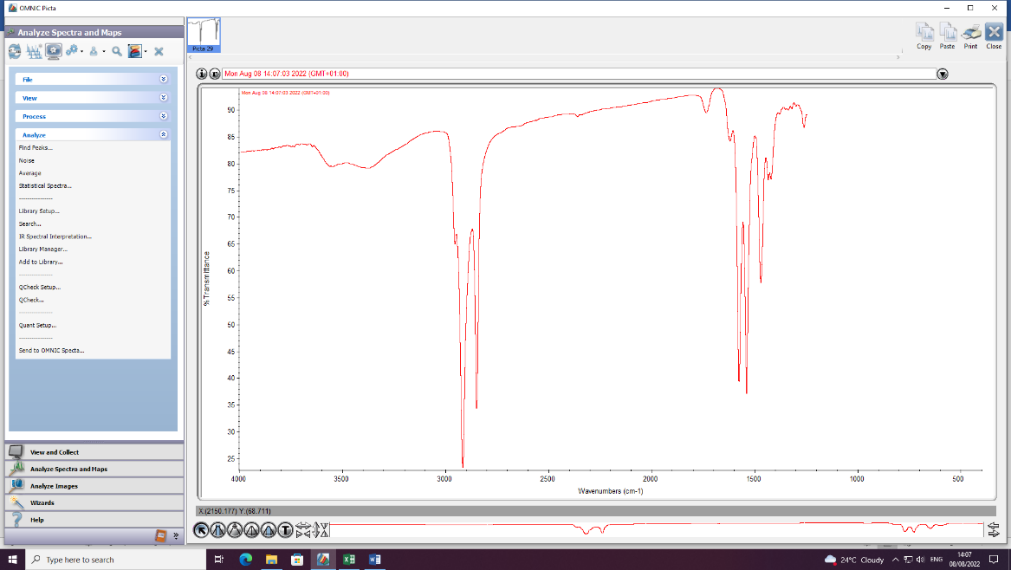


1000

3000

4000

200011

90

80

70

60

50

40

30
