# Supplementary material for: Microplastics in cardiopulmonary bypass: quantification and characterization of particles across systems
Source: Interdiscip Cardiovasc Thorac Surg. 2025 Jun 10;40(6):ivaf080. doi: 10.1093/icvts/ivaf080 (PMC12161987; doi:10.1093/icvts/ivaf080)
Supplement: ivaf080_Supplementary_Data [file ivaf080_supplementary_data.zip › Supplementary Table ST1.docx]

**Supplementary Table 1.** CPB circuit sample information alongside the number of MPs identified within samples by μFTIR spectroscopy. The most abundant polymer types (for each circuit type) and three different contamination adjustments are used to display results in units of MP/L of Hartmann’s solution. Abbreviations; LOD, limit of detection; LOD, limit of quantification; MiECC, minimal invasive extracorporeal circulation; MP, microplastic; PDMS, poly-dimethyl siloxane; PBMA, poly N-butyl methacrylate; PDMA, poly decyl methacrylate; PP, polypropylene; PA, polyamide (nylon); PMAA, poly N-methyl acrylamide; PE, polyethylene; SR, silicone rubber; SP, silicone polymer.

| **Circuit type** | **Circuit Run** | **MP polymer** | **Unadjusted value**  **(MP/L/h^-1^)** | **Blank subtracted value**  **(MP/L/h^-1^)** | **LOD/LOQ Value**  **(MP/L/h^-1^)** |
| --- | --- | --- | --- | --- | --- |
| Conventional | 1 | PDMS  PBMA  PDMA  SR  SP  PP  PMAA | 32  13.3  5.3  4  8  1.3  1.3 | 31.7  13.3  5.3  4  8  1.3  1.3 | 31.7  13.3  5.3  4  8  1.3  1.3 |
| Conventional | 2 | PBMA  PDMA  PE | 5.3  49.3  4 | 5.3  49.3  4 | 5.3  49.3  4 |
| Conventional | 3 | PDMS  PBMA  PDMA  SR  SP | 26.7  20  1.3  2.7  8 | 26.3  20  1.3  2.7  8 | 26.3  20  5.3  2.7  8 |
| MiECC | 1 | PBMA  PDMA  PP  (PA)  PE | 1.3  1.3  1.3  1.3  12 | 1.3  1.3  1.3  1.1  12 | 1.3  1.3  1.3  -  12 |
| MiECC | 2 | PP  (PA)  PE  PMAA | 12  5.3  10.7  6.7 | 12  4.4  10.7  6.7 | 12  -  10.7  6.7 |
| MiECC | 3 | PBMA  PDMA  PP  (PA)  PE | 5.3  2.7  36  14.7  9.3 | 5.3  2.7  36  13.9  9.3 | 5.3  2.7  36  -  9.3 |
